# Understanding Family Experiences: A Study on Mental Health Literacy in Adolescent Eating Disorder Diagnoses

**DOI:** 10.3390/nursrep14040302

**Published:** 2024-12-20

**Authors:** Eva García Carpintero-Blas, Esperanza Vélez-Vélez, Esther Martínez-Miguel, Alberto Tovar-Reinoso, Pablo Del Pozo-Herce, Carlos González-Navajas, Cristina Gómez-Moreno

**Affiliations:** 1 NBC Group, Health Department, School of Life and Nature Sciences, Nebrija University, 28248 Madrid, Spain; egarcibl@nebrija.es (E.G.C.-B.); emartinezmi@nebrija.es (E.M.-M.); 2 Research Group on Innovation in Health Care and Nursing Education (INcUidE), University of UNIE, 28040 Madrid, Spain; alberto.tovar@universidadunie.com; 3 School of Nursing, Fundación Jiménez Díaz, Madrid Autonomous University, 28049 Madrid, Spain; evelez@fjd.es (E.V.-V.); cgomezm@fjd.es (C.G.-M.); 4 UNIE Universidad, 28040 Madrid, Spain; 5 Child and Adolescent Psychiatry and Psychology Service, Child Hospital Niño Jesus, 28009 Madrid, Spain; cgnavajas@salud.madrid.org

**Keywords:** family therapy, nursing, eating disorders, qualitative research, mental health literacy

## Abstract

Background: Adolescent eating disorders pose a significant public health challenge and strongly affect both physical and emotional well-being. Early diagnosis is important for improving treatment outcomes, though it remains complex due to multiple influencing factors. The family perspective is essential in this process, as it provides valuable insights into changes in adolescents’ habits and emotional health. Methods: A descriptive qualitative study was conducted between January and February 2023. Interviews were conducted with 12 participants using a phenomenological approach to explore the experiences and perspectives of family members of adolescents with eating disorders. Results: Four thematic blocks comprising several categories were identified: (T1) diagnosis, (T2) family, (T3) resources, and (T4) treatment. The participants emphasized the need for early intervention and a multidisciplinary approach to the treatment of eating disorders. Family therapy was recognized as vital to treatment, and common dissatisfaction with the public health structure underscores the need for greater investment in research and access to specialists. Mental health nurse practitioners play an important role in providing comprehensive care and support, as well as mental health literacy. Conclusions: A holistic, patient-centered approach, including family involvement and appropriate support systems, is key to improving the outcomes and quality of life of adolescents undergoing treatment for eating disorders. Improving healthcare resources and addressing the challenges faced by families is essential. In addition, mental health literacy is critical, as it enables families to understand the disease better, make informed decisions, and actively participate in the recovery process, thus contributing to improved therapeutic outcomes and quality of life for patients.

## 1. Introduction

Eating disorders (EDs) are prevalent psychiatric illnesses that usually manifest during adolescence, a critical stage of neural development, physical and psychological growth, and self-exploration [1,2]. This period of life, marked by significant physical, emotional, and social changes, makes adolescents particularly vulnerable to developing these disorders, which profoundly affect their relationship with food, body image, and overall well-being [3].

According to the latest update of the *Diagnostic and Statistical Manual of Mental Disorders* (DSM-5-TR) published in 2022 [4], the main eating disorders include anorexia nervosa (AN), bulimia nervosa (BN), and binge eating disorder (BED). AN is defined by the extreme restriction of energy intake, resulting in a significantly low body weight, an intense fear of weight gain, and a distorted perception of one’s body image. For BN, the diagnostic criteria include recurrent episodes of binge eating, where the person consumes an excessive amount of food in a short period, followed by inappropriate compensatory behaviors, such as self-induced vomiting, laxative use, or excessive exercise, occurring at least once a week for three months. BED is characterized by recurrent episodes of uncontrolled food intake, accompanied by significant emotional distress, without the presence of compensatory behaviors, and must also occur at least once a week for a minimum period of three months [4]. Recent advances have linked anorexia nervosa (AN) symptoms such as hyperactivity and food obsession to hypoleptinemia, a biological response to fat loss [5]. This insight can improve families’ understanding by showing that these behaviors are biological, not deliberate. Enhanced empathy may strengthen family support, aligning with their crucial role in treatment.

The prevalence of EDs has increased significantly in recent decades [6,7] and further exacerbated by the COVID-19 pandemic [3]. During COVID-19, an increased incidence of restrictive eating behaviors, binge eating, and excessive exercise was reported in patients with eating disorders [8]. A recent review of epidemiological studies confirmed that the incidence of AN is increasing in young individuals worldwide, especially in those under 15 years of age, with a peak age of onset between 13 [9] and 18 years [10]. These disorders differ in their onset [11], with AN manifesting at younger ages than BN [12]. The lifetime prevalence of AN is 4% in the female population and 0.3% in the male population. The lifetime prevalence of BN is up to 3% in the female population and more than 1% in the male population, with symptoms increasing among girls aged 14 to 16 years and among boys aged 16 years and older [9,10].

The etiopathogenesis of EDs is multifactorial, involving both genetic and environmental factors [3]. Sociocultural influences, such as social pressure to conform to beauty standards, the impact of social media, and the overvaluation of thinness, exacerbate these disorders, making adolescents more vulnerable to dysfunctional eating behaviors [3,13]. Early diagnosis and appropriate intervention are necessary to improve prognosis and reduce associated risks, such as severe malnutrition, physical complications, and chronic emotional problems. Given their prevalence, severity, and tendency to become chronic, EDs represent a serious public health issue requiring specialized treatment [14]. In this context, mental health literacy (MHL) is essential for effective ED management. This concept is defined as the ability to obtain, process, and understand the knowledge and skills necessary to promote mental health [15]. For ED patients and their families [16,17], adequate MHL is essential for understanding the nature of the disorder, the importance of timely treatment, and how to meet the challenges of recovery, ultimately improving both physical and psychological outcomes [18,19,20].

Equally important is the role of the family, which is a key pillar in eating disorder (ED) care, especially in observing the early signs of the disorder and providing support throughout diagnosis, treatment, and recovery [21]. A supportive family environment and active involvement positively influence treatment outcomes [22] and are important both at home and in the hospital setting. However, for families to provide effective support, they need information, emotional help, and tools to manage these complex diseases. In this regard, nursing professionals play a key role, acting as intermediaries in identifying symptoms, providing ongoing support, and educating families on how to manage these disorders [23,24]. In hospital settings, nurses are fundamental in the implementation of therapeutic strategies and in the follow-up to prevent relapses. However, in other settings, such as primary or outpatient care, the role of nurses is complemented by other health professionals, such as physicians, psychologists, and nutritionists. As part of multidisciplinary teams, nursing professionals facilitate communication between adolescents, their families, and other healthcare professionals, ensuring a comprehensive and collaborative approach to treatment [23,25].

In this study, the authors sought to explore family perspectives on the diagnosis of adolescent EDs by applying a qualitative approach to understanding the experiences, challenges, and emotional needs of families in this process. In providing these insights, the aim is to highlight the importance of a holistic approach, where families and nursing professionals work together to improve outcomes in the treatment of adolescent EDs.

## 2. Methods

### 2.1. Study Design

A descriptive qualitative study based on the theoretical/methodological orientation of phenomenology was conducted to explore the experiences of family members of adolescent patients diagnosed with EDs [26]. Phenomenology, which aims to understand the essence of a phenomenon through first-person-narrated experiences, provides the framework for obtaining a detailed description of a family’s lived experiences [27]. This study was conducted in accordance with the Consolidated Criteria for Reporting Qualitative Studies (COREQ) and the Standards for Reporting Qualitative Research [28] (see Supplementary File S1).

### 2.2. Experience and Role of Researchers

The research team consisted of four women and three men, including four nurses with experience in qualitative research design (E.G.C.-B, E.M-M, A.T-R, and C.G-M) and two researchers with clinical and research experience in mental health (P.D.-H and C-G.-N). Data were triangulated by two external researchers (E.V-V and C.G.-N). None of the research team members had prior relationships with the participants. The theoretical framework for this qualitative research, as well as the researchers’ viewpoints and their reasons for conducting the study, were defined before starting the research [28].

### 2.3. Participants and Setting

A snowball-based, purposive sampling approach [29] was employed to recruit relatives of adolescents aged 12 to 19 years diagnosed with EDs. Participants who had not lived with the patient since the diagnosis and those whose family members were diagnosed less than one year ago were excluded. Relatives were recruited through posters displayed in a social-health center and posts on social media. In addition, adolescents diagnosed with EDs were initially identified and contacted through the health team involved in their care. With the consent of the adolescents, the health team facilitated contact with their relatives. To improve the snowball-based, purposive sampling method, participants were encouraged to recommend other relatives in similar situations who might be interested in participating in the study. Data saturation was reached at participant number twelve when further coding was no longer feasible with additional new information [30]. Therefore, the researchers did not seek to recruit more participants. No dropouts occurred during the study. Table 1 shows the demographic data.

### 2.4. Data Collection Instrument

Data were collected between January and February 2023 through in-depth interviews and field notes. These interviews were conducted in a semi-structured manner, following a question guide designed to explore specific topics of interest (Table 2).

Semi-structured in-depth interviews were used as the primary data collection method. A question guide based on the existing literature (Table 2) facilitated an in-depth exploration of participants’ thoughts and experiences of the phenomenon [29]. Its flexible nature allows participants to express themselves freely while the researcher probes into areas of interest [31]. Individual interviews were conducted by two researchers (E.G.C-B and C.G-M) either face to face at the preferred place and time or via video call, depending on the participant’s preferences. Due to the topic’s sensitive nature, family members were informed that they could stop the interview at any time if they found it emotionally distressing. On average, each interview lasted approximately 57 min. Field notes were also collected by the researchers, providing additional insights into participants’ personal experiences and behaviors during data collection, as well as the researchers’ reflections on methodological aspects [32]. The transcripts were returned to the participants for additional comments, and all transcripts and field notes were securely stored in a digital location with restricted access.

### 2.5. Data Analysis

A qualitative analysis of each interview and the researchers’ field notes was performed using an inductive thematic approach [33]. Codes were generated to identify the most descriptive content, which was then reduced and grouped to identify common categories representing significant content units. This process led to the emergence of thematic areas describing the experiences of the study participants. Double and independent coding was carried out by five researchers (E.G.C-B, C.G-M, A.T-R, E.M-M, and P.D.-H) on each interview and each field note. They then met to discuss, compare, and refine their findings. Subsequently, the same process was carried out with the themes. In addition, joint meetings were held to consolidate the analysis results and represent the parents’ experiences, and an external audit was performed by an independent researcher to ensure validity. All coding processes were discussed by the research team until a consensus was reached on the main categories and themes, creating a final matrix of categories. The computer program ATLAS-TI-24 was used for the analysis [34].

### 2.6. Quality and Rigor Criteria

This study was conducted in accordance with the Consolidated Criteria for Reporting Qualitative Research [28] and the Standards for Reporting Qualitative Research [35]. The rigor and reliability of the qualitative data were ensured by applying the criteria of Guba and Lincoln (see Table 3).

### 2.7. Ethical Considerations

The study was conducted according to the Declaration of Helsinki and received ethical approval from the Research Ethics Committee of the University Hospital (PIC015-23FJD). All participants were informed of the objectives of the study and signed a written informed consent form before the interviews, assuring them that they had the right to withdraw at any time without consequences. The interviews were conducted anonymously, voluntarily, and confidentially, without collecting personal data or devices that could identify the informants, and were recorded with the permission of the participants and then transcribed verbatim. The information obtained was treated anonymously and confidentially, complying with the General Data Protection Regulation (EU) 2016/679 of the European Parliament and Organic Law 3/2018. The researchers did not report any ethical, moral, or legal conflicts, nor did they receive financial compensation, just as the participants did not receive compensation for their collaboration in the study.

## 3. Results

Twelve individual interviews were conducted with family members of patients with eating disorders. More than half (58.33%) were female interviewees, with an average age of 44.7 years. Of the patients in question, 75% were diagnosed with AN, while 25% were diagnosed with BN, with an average age at diagnosis of 17.4 years.

### 3.1. Themes

Four thematic blocks comprising several categories were identified: (T1) diagnosis, (T2) family, (T3) resources, and (T4) treatment (see Table 4).

### 3.2. Theme 1: Diagnosis

During the interviews, the participants shared their perspectives on possible triggers for ED development in their family members and the circumstances surrounding the onset of the disorder. Some mentioned the influence of social media and comparisons with peers as influential factors.

“*My relative compared herself to girls her age, questioning why they could eat without gaining weight, while she felt that she gained weight even when eating minimal amounts of food.*”

E6

Additionally, traumatic events, such as the death of a loved one, were highlighted as possible triggers for ED:

“*My relative faced a difficult emotional circumstance after her father’s death. She had a significant bereavement problem, which led her to self-harm and injure her wrists.*”

E2

Confinement due to the COVID-19 pandemic was not considered a triggering or aggravating factor, but it did complicate the situation experienced, and in some cases, this led many families to realize that their adolescents had an ED.

“*Well, of course, you see yourself locked up at home without being able to go out, without being able to do sport, locked up like a family therapy beast everything. We all eat lunch, breakfast, and dinner at the same time and there is no way to escape.... So, yes, it was very traumatic then because she didn’t want to leave the room, she cried...*”

E2

The participants also identified personality traits such as high levels of control and low self-esteem as influential factors in ED-related behaviors.

“*She exercised, she went to the gym a lot, she is a very active person, very controlling, if she leaves something somewhere she wants to see it there, she has everything very controlled.*”

E10

Some agreed that low self-esteem was an underlying cause of their relatives’ illness. In their opinion, an ED could manifest as a problem related to self-acceptance and self-esteem, including perfectionism. For them, the ED represents a more profound problem beyond the simple relationship with food.

“*(...) but it is not about being fat or thin; it is a deeper problem of accepting yourself as you are, that is to say, that the manifestation is food, but the origin is something else.*”

E11

They also noticed significant changes in the behavior of their loved ones:

“*She used to be a cheerful and outgoing adolescent, but as a result of this whole process, she became defensive, bad-tempered, overreacting to any comment and even crying. The situation became complicated for everyone.*”

E6

The situation became complex for everyone involved, as these emotional and behavioral changes profoundly impacted family dynamics. Furthermore, the family members noted that their affected loved ones exhibited emotions and behaviors characterized by isolation, avoidance, and frequent bouts of bad moods. They described a shift in the individual’s responses and noted that they became more defensive:

“*I have always kept joking, and she has always played along with me... but I remember once I made a comment about her and her reaction was to start crying inconsolably. Apart from that, she was continually defensive.*”

E7

When asked about their initial reaction to receiving their relative’s diagnosis and its impact, more than half of the participants said that they did not experience any significant change in their emotions. This lack of surprise was attributed to their pre-existing suspicions about the condition. One family member expressed this sentiment by saying,

“*Mmm, I did not feel any special way because I already suspected it.*”

E9

They noticed certain signs and symptoms that led them to anticipate the possibility of an eating disorder.

However, there were participants who, despite having some suspicions, still expressed surprise at the formal diagnosis. They acknowledged that they were expecting the news, but the severity of their relative’s health situation surpassed their expectations. One participant explained,

“*Well, the truth is that I was surprised because we were waiting to be told that, but I did not think it was or she was in such a serious moment.*”

E4

This unexpected seriousness added an extra layer of complexity and concern to their emotions.

Furthermore, some family members admitted feeling confused in response to the diagnosis. They were unsure about how to handle the situation and what steps to take. The gravity of the situation and the unfamiliarity of dealing with an eating disorder led to a sense of uncertainty and unease. One participant shared their experience, saying,

“*I felt confused at the time of diagnosis because I did not know what to do...*”

E7

The lack of clarity in understanding the disorder and its implications added to their emotional challenge in coping with the diagnosis.

Overall, the reactions to the diagnosis varied among family members, with some confirming their suspicions, others being surprised by the severity, and a few feeling unsure and confused about how to proceed. These emotions reflected the complexity of facing an eating disorder diagnosis within the family unit.

### 3.3. Theme 2: Family

First, we explored family structure and relationships, identifying both families with traditional, stable structures and those with more complex family situations. One participant described his family as a traditional nucleus, consisting of parents and a sibling, while another noted that, after the death of his father, he experienced problems at home, which led him to want to leave home.

“*We are family, the family nucleus in this case is structured, his father and I are together, and we have another son...*”

E1

“*His father had died [...], he began to have a lot of problems at home, with his mother, he did not want to be with her, he wanted to leave home.*”

E8

Regarding the pre-existing relationship with their relative before the illness, all participants reported having a good relationship characterized by trust and open communication. There were no instances of problematic relationships before the disorder emerged, as most participants described it as a typical and healthy family dynamic.

“*A normal relationship, of trust, of asking for your opinion, advice... I mean, each adolescent is different, but it was a normal relationship.*”

E12

Interestingly, in one case, the illness seemed to have strengthened the family bond, making them feel more united and resilient:

“*No, it didn’t change, it hasn’t changed. Not at all, it has strengthened; on the contrary, it has made us stronger.*”

E1

However, for most participants, the illness had a negative impact, leading to a breakdown in trust and a less intimate relationship. They attributed this change to the necessity of lying that often accompanies the development of the disorder.

“*To act on the illness you need to lie, don’t you? And then, of course, everything changed.*”

E9

The impact of the illness extended beyond family relationships and permeated various aspects of daily life. Family members shared experiences of adjusting their routines and habits to accommodate the challenges posed by the illness. For some, holidays became stressful, as they had to navigate unfamiliar territories and disrupted routines. Additionally, the marital relationship was heavily affected, causing disagreements and conflicts over the best approach to support their loved one’s recovery:

“*I had to change my daily life.*”

E4

“*The first year of holidays I remember it as a nightmare, we took her out of her comfort zone and it was chaotic.*”

E6

“*It worsened my relationship with my wife.*”

E7

“*We had many problems within the family nucleus, as my wife and I did not agree on the best way for her to be cured. We argued every day, we cried out of helplessness and although our aim was the same, we could not agree.*”

E7

“*Above all, we saw that it was fundamental to be well in the marriage. That was paramount. And then, if you are well, you can help your children, but if not, you can’t.*”

E3

In several cases, family members took on new roles, with siblings becoming caregivers. This change in role brought about feelings of being overwhelmed, hopeless, and unsure of how to handle the situation. Some expressed frustration when their relative refused help, leaving them feeling powerless and desperate to assist.

“*She changed with his siblings, because also siblings at the beginning tend to.... to watch over... and the role of siblings is not that.*”

E4

“*You see that a person is drowning and you throw them a float, and they don’t catch it.*”

E4

Furthermore, the slow progress of recovery from the illness added to the uncertainty and anxiety experienced by family caregivers. Eating disorders are often unpredictable, and not knowing what to expect or how to effectively support their loved one further compounds the burden that family members carry.

“*Feeling hopeless... hopeless, I didn’t see myself as capable.*”

E7

Despite the challenges, family involvement in the treatment process was highly valued and considered vital to the patient’s recovery. Family members expressed a genuine interest in actively participating in their relative’s treatment, often suggesting joint sessions or seeking educational resources to enhance their support, emphasizing the importance of family therapy and support in the recovery journey:

“*(…) there were courses for families on how to deal with Christmas, meetings… and, believe me, that was fundamental to me and I truly believe, it was very important for her also.*”

E2

Most participants felt deeply involved in the treatment and considered their contribution highly valuable. Some expressed satisfaction with their involvement, while others expressed a strong sense of commitment.

### 3.4. Theme 3: Resources

All participants unanimously agreed that the public health system has scarce resources to effectively address the complexities of this type of pathology. Although they initially sought help from public services, none of their relatives received treatment there. They attributed this issue to the overwhelming number of patients and the lack of adequately trained staff, resulting in a saturated system and the experience of what is known as scarce public resources.

“*The public health system, in my opinion, has very few resources for this type of patient.*”

E7

Participants highlighted the sluggishness of the services, including long waiting times for appointments and consultations, which hindered effective follow-up and continuity of care:

“*Because of social security, they offered us a psychologist almost for the following year.*”

E12

Although they expressed satisfaction with the treatment and resources available during hospitalization, they emphasized that such care was only temporary at the hospital level and did not extend to outpatient settings, creating a gap in the long-term management of the disorder:

“*(…) Yes, during her hospitalization, yes… she was very grateful because all the professionals in the hospital helped her a lot, they were great, but after that... mmm... I think it was not enough, she did not have a proper follow-up.*”

E5

Moreover, they raised concerns about the lack of resources and support available for patients who transitioned to adulthood and were no longer eligible for pediatric treatments. According to their perception, resources for adults were less comprehensive than those available for adolescents, and searching for appropriate resources was challenging upon reaching adulthood.

When seeking therapies or consultations with specialists in the field, they discovered that the options were predominantly in private health services, leaving them to navigate this situation on their own, which incurred additional costs:

“*She has been managing it privately, she has had to struggle and look for help on her own.*”

E4

Based on their experiences, the participants agreed that an effective treatment approach should involve a multidisciplinary team comprising psychologists, psychiatrists, nutritionists, mental health nurse practitioners, and other professionals. However, this comprehensive approach is hindered by the high costs of such services, making them inaccessible to many individuals and families:

“*I was shocked at the prices charged by each one, there are few of them, and on top of that, they are very expensive privately.*”

E7

The role of nursing in the treatment process was highly regarded by those who had had direct contact with mental health nurse practitioners, especially during hospitalization, where they had more contact with them, as well as in outpatient follow-ups:

“*Nursing professionals have an essential role in the approach.*”

E4

However, some participants admitted that they were not fully aware of the specific role of mental health nurse practitioners in this area, as they had not had direct contact with nursing professionals during the course of the disease:

“*Honestly, I don’t know because... surely yes, it is possible that nursing plays an important role, but so far we have not had the opportunity to learn about it.*”

E7

Others recognized the significance of nurses working in parallel with doctors, emphasizing the importance of continuous and individualized care, which includes regular follow-up through calls or consultations, complementing medical treatment:

“*It is very important that the doctor sees you, but the follow-up by the nurse…*”

E1

### 3.5. Theme 4: Treatment

The vast majority of family members currently acknowledge that they would approach the situation differently based on their learning and experiences. As one participant aptly stated,

“*Experience is the mother of science*.”

E2

They unanimously agreed on the importance of timely intervention in treating the illness, emphasizing the need to act swiftly without dismissing the possibility of the disorder, even in seemingly idyllic situations:

“*Act quickly and don’t keep thinking that it can’t be because your daughter is idyllic... because you think that it can’t happen to her and it does happen, and that ends up prejudicing the diagnosis of her illness.*”

E6

Over time, participants gradually became more aware of the challenges that they were facing and had to confront, prompting them to seek assistance and coping strategies through various channels, ranging from primary care to the hospitalization of their relative. To address the complexities of the illness, family members embraced a multidisciplinary approach, seeking assistance from various professionals. They acknowledged the significance of incorporating family members into the therapeutic process, recognizing their role as essential caregivers:

“*I think this help could be improved by doing family therapy, group therapy, or including family members in some kind of consultation or information session... to include them in the treatment as they are going to be the main caregivers.*”

E4

Despite this recognition, it was noted that none of the participants’ relatives had received family-based therapy.

When asked about potential improvements to the healthcare resources for EDs, the responses varied. However, they unanimously agreed on the necessity of increasing the number of professionals to lower the doctor/nurse–patient ratio and ensure quality care with adequate time allocation for each individual’s needs.

“*I would like everything to be easier and quicker.*”

E6

They emphasized the importance of personalized attention, akin to treating other medical conditions where tailored treatment is provided.

“*Like when you have a cold and they prescribe paracetamol, just the same.*”

E1

Moreover, given the significant impact of this issue on so many individuals, the participants underscored the need for increased investment and research in EDs. They acknowledged the scarcity of information about these disorders and believed that further research could lead to the development of alternative and more effective treatment methods.

“*I’ve read that it’s what we know the least about; maybe they need to do more research.*”

E5

**Figure 1:**
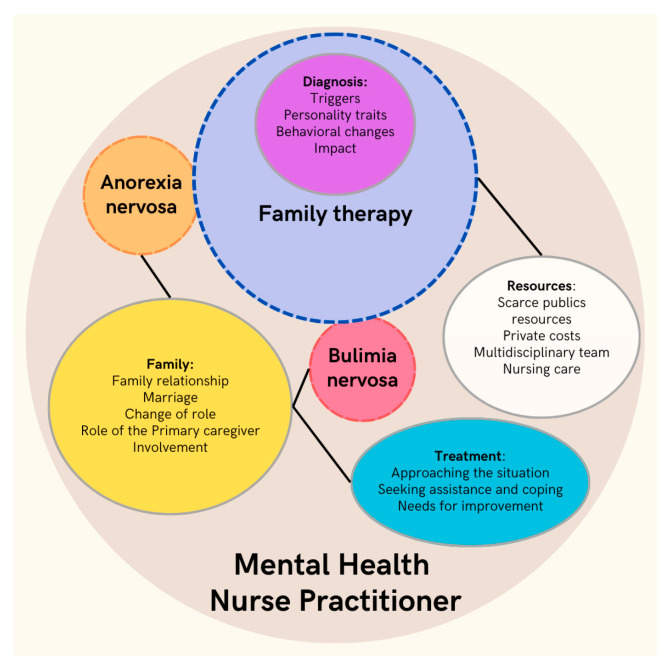
Map of agents and interactions.

These results illustrate the complexity of families’ perceptions and experiences of adolescent eating disorders. Figure 1 shows a map of agents and interactions and allows us to establish the following themes: The first theme involves the diagnosis (triggers, personality traits, behavioral changes, and impact). The second theme deals with the family (family relationship, marriage, change of role, role of the primary caregiver, and involvement). The third theme deals with resources (scarce public resources, private costs, multidisciplinary teams, and nursing care), and the fourth theme deals with treatment (approaching the situation, seeking assistance and coping, and needs for improvement). All of these are related to each other and to eating disorders. The ATLAS-TI program was used to code and synthesize the data, and a graphic designer prepared the results in the form of a map of agents and interactions, as shown in Figure 1.

Figure 1 presents a map of agents and interactions that synthesizes the main findings of the study, highlighting the relationships between the four identified themes and their connection to eating disorders (EDs). These themes are (I) diagnosis, which covers triggers, personality traits, behavioral changes, and the impact of the diagnosis; (II) family, which explores family dynamics, role changes, the role of the primary caregiver, and involvement in treatment; (III) resources, which details limitations in public resources, private costs, the need for a multidisciplinary team, and the role of nursing; and (IV) treatment, which addresses the initial approach, coping strategies, and needs for improvement in services. All these themes are interrelated: for example, the diagnosis impacts the family dynamics, the perception of available resources, and treatment strategies. The graphic representation uses circles and arrows to illustrate these relationships, highlighting the interaction and mutual influence between the factors, facilitating a comprehensive understanding of the families’ experience, and integrating the different elements that make up the management of EDs.

## 4. Discussion

EDs are complex diseases influenced by biological, environmental, social, psychological, and cultural factors [36,37]. This study highlights the importance of family perspectives in understanding how these factors interact in the diagnosis and treatment processes. Among the most relevant findings, the family members identified triggers, such as low self-esteem and the impact of social networks, that promote unrealistic beauty ideals. In addition, the COVID-19 pandemic exacerbated these risk factors, revealing previously unidentified eating behavior problems in some patients.

A central theme in this study is the role of the family as the primary caregiver. The participants expressed feelings of helplessness, overload, and hopelessness, in line with previous research evidencing the emotional impact of these responsibilities on family members’ quality of life [38,39]. This analysis of family responses provides valuable insight into how they perceive the effects of the ED on their personal experience, family dynamics, and their interaction with the healthcare system. For example, fathers often expressed a greater sense of helplessness compared to mothers, whereas sisters emphasized changes in sibling relationships and roles assumed within the family. This diversity of perspectives could enrich the understanding of the needs and challenges of families as a whole, allowing the design of more comprehensive intervention strategies tailored to the reality of each member.

An important finding of the study is the need to integrate the entire family into the patient’s recovery process. EDs affect not only the individual sufferer but also the family dynamics, generating tensions, feelings of guilt, and misunderstanding. Involving family members in therapy can address these challenges, provide education about the nature of the disorder, and empower them to be active partners in recovery [40,41]. This involvement may also identify dysfunctional patterns in communication or behavior that perpetuate the problem, and addressing these issues promotes positive changes within the family.

Mental health literacy is a key aspect in helping families understand EDs and effectively support patients. In the same vein, the NICE clinical practice guideline supports adult caregivers through appropriate programs, information, and psychosocial support [42]. However, the results show that many participants lacked sufficient information about the disorder and did not know how to collaborate with the healthcare team. Improving the level of MHL could empower families, reduce the stigma associated with these disorders, and encourage greater adherence to treatment [16,18,19,20,24]. In addition, recent advances in the biological understanding of EDs, such as the role of hypoleptinemia in anorexia nervosa [43], underscore the importance of integrating biological knowledge into educational strategies aimed at family members [44]. This holistic approach could strengthen collaboration between patients, families, and healthcare professionals.

Widespread family dissatisfaction with available resources was a recurring theme. The limitations of the public health system, the need to use private services, and difficulties in the transition from pediatric to adult care are significant barriers to ensuring a continuum of quality care [39,45,46]. The participants highlighted the need for a multidisciplinary and individualized approach that includes physicians, psychologists, nutritionists, and specialized nurses. In this regard, nurses not only impart quality care but also promote informed decision making [47]. In addition, nurses help patients develop skills to recognize harmful behaviors and thoughts, teaching them to make healthier dietary and self-care decisions, such as identifying warning signs, which is important for restoring balanced eating patterns, together with the multidisciplinary team [17,18,20,24,47].

Providing family members with clear and accessible information about the biological aspects of the disorder not only facilitates their understanding but also provides them with the necessary tools to participate more actively and effectively in the treatment process. Along these lines, the treatment of EDs should evolve to integrate the most recent advances in biology, such as the findings on hypoleptinemia in AN, according to Hebebrand et al. (2024) [43]. This biological knowledge provides a deeper understanding of the physical basis of the disorders, which can significantly improve mental health literacy strategies aimed at families. By understanding that EDs have both biological and psychological roots, the stigma associated with these disorders can be reduced, fostering a more collaborative and empathetic environment both within the family and in the healthcare setting [43]. Several studies have highlighted widespread dissatisfaction among family members regarding the care and resources available for EDs [39,45]. The transition from care in adolescence to care in adulthood poses challenges, resulting in a significant decrease in available resources upon reaching adulthood [46]. To ensure the continuity of care, a multidisciplinary and individualized approach is essential for meeting the demands expressed by the family members interviewed who sought comprehensive care with diverse perspectives [38,44].

The importance of nursing in the management of EDs, especially the critical role of mental health nurses, has been highlighted by various sources [48,49]. These patients often present complex challenges that require staff with specific expertise. Nurses play a key role in health education, promoting self-care, patient autonomy, and healthy eating habits, making them indispensable members of multidisciplinary teams [48,49,50]. This study revealed that, although many families were not fully aware of the extent of the nurses’ roles, they perceived their contributions as essential in the care of their loved ones. It highlighted the close relationship that these professionals develop with patients, facilitating a patient–professional bond that is valuable in providing effective care, emotional support, and continuous follow-up throughout the treatment process. This involvement not only improves clinical outcomes but also empowers patients to take charge of their well-being, promoting positive lifestyle changes and better adherence to treatment [17,18,19,49,50].

However, the role of nurses should be understood as part of a comprehensive, multidisciplinary approach that also requires the active inclusion of families in the recovery process. Collaboration between multidisciplinary teams and the continuity of care, especially in the transition from pediatric to adult care, are crucial for optimizing outcomes for patients with EDs. In addition, addressing the challenges faced by family caregivers by providing adequate support and resources is essential for the effective management of these complex diseases. Increasing investment in research and public healthcare resources is equally imperative to meet the growing demand for specialized treatment and ensure comprehensive care for both patients and their families [16,20].

### 4.1. Strengths and Limitations

This study provides valuable insight into the experiences and perceptions of family caregivers of individuals with eating disorders. Through its qualitative approach, a comprehensive and in-depth understanding of the challenges and needs faced by these families is achieved. By considering the views of diverse family members and health professionals, the study provides a multidisciplinary perspective on the treatment of eating disorders, enriching the overall understanding of the topic. In addition, the study provides practical recommendations, such as the importance of family therapy and the key role of nursing in treatment. These suggestions may inform future treatment protocols and interventions for eating disorders.

This study has limitations in that further research with larger and more diverse samples is needed to validate the results in different populations. The small sample size and possible lack of diversity among participants may limit the generalizability of the findings. Because the study relied on family members’ accounts of their experiences, there is the potential for recall bias. Family members may not accurately recall all events or may perceive them differently from how they occurred. In addition, the qualitative nature of the data collected may introduce subjectivity into the interpretation of participants’ responses. It is essential to ensure rigor in the analysis and interpretation of the data to minimize possible biases.

In addition, the method adopted in this study, which consisted of interviewing individual family members, proved to be highly productive in allowing an in-depth analysis of their personal perspectives and experiences. However, this approach did not directly capture the dynamics of family interactions, a central aspect of most family therapy interventions. These interactions may reveal patterns of communication, conflict, and support that significantly influence the management of eating disorders. Future research might consider incorporating methods that explore family interactions, such as group interviews or observational sessions, to complement individual findings and provide a more holistic view of the family experience in this context.

### 4.2. Nursing Implications

This study highlights the importance of adopting a personalized therapeutic approach for patients with eating disorders, emphasizing the need for healthcare professionals, such as psychologists, psychiatrists, nutritionists, and nurses, to work in a multidisciplinary team to address the multifactorial causes of these disorders. This approach will ensure that patients receive comprehensive and individualized care, promoting improved treatment outcomes. In addition, the research highlights the critical role of family members in the treatment process, indicating that healthcare professionals should actively involve families in therapy sessions, support groups, and educational programs to improve patient care. Recognizing and addressing family dynamics can contribute significantly to the patient’s recovery and overall well-being.

Likewise, the dissatisfaction expressed by family members about the insufficiency of public resources for the treatment of eating disorders requires urgent attention, and health authorities should invest in increasing the availability of resources and specialized personnel to provide timely and quality care. Mental health nurse practitioners play a key role in the treatment of eating disorders by building strong therapeutic relationships and promoting self-discipline and healthy eating habits. Patient empowerment is another area where MHL and nursing intertwine; nurses teach self-care skills that enable patients to take control of their recovery, including recognizing relapse symptoms and planning healthy meals.

In addition, they play a central role in the coordination of multidisciplinary care by ensuring that medical indications are translated into understandable terms for patients and families, which facilitates greater adherence to treatment. Finally, nurses are key in the prevention and early detection of eating disorders, as they can intervene in community or school settings to promote MHL at early stages, enabling people to recognize the initial signs of an ED in themselves or others, which contributes to early diagnoses and more effective treatment.

## 5. Conclusions

In conclusion, our study shows the complexity of EDs, which have no single trigger, highlighting the need for personalized and tailored care to achieve effective therapeutic outcomes. EDs affect not only the individual but also the family dynamics, underscoring the importance of involving families in the treatment process, both to support the patient and to address the significant psychological implications faced by caregivers. Dissatisfaction with public resources highlights the urgency of increasing the availability of specialized personnel and improving health system services. In this context, MHL emerges as an essential component, with nursing professionals being a fundamental pillar in providing care, emotional support, and psychoeducation to family members, in addition to coordinating patient follow-up, together with the multidisciplinary team. These interventions not only improve the prognosis of patients but also contribute to preventing relapses and promote a better long-term quality of life for patients and family members.

Future studies should evaluate the efficacy of family therapy in the treatment of EDs and explore the impact of increased investment in public health and in the recruitment of specialized professionals. This more holistic approach, combining family support, MHL, and a strengthened health system, has the potential to generate more effective treatments and provide significant benefits for both patients and their families.

## Figures and Tables

**Table 1:** Participant characteristics (*n* = 12).

Participant	Sex of Interviewee	Age of Interviewee	Relationship	ED Diagnosis	Age at Diagnosis	Adolescent Gender
E 1	Woman	56	Mother	Anorexia nervosa	19	male
E 2	Woman	51	Mother	Anorexia nervosa	17	female
E 3	Man	54	Father	Anorexia nervosa	17	female
E 4	Woman	21	Sister	Anorexia nervosa	18	female
E 5	Man	75	Grandfather	Bulimia nervosa	17	female
E 6	Woman	49	Mother	Anorexia nervosa	17	female
E 7	Man	50	Father	Anorexia nervosa	17	female
E 8	Woman	25	Sister	Bulimia nervosa	18	female
E 9	Woman	54	Mother	Anorexia nervosa	17	female
E 10	Man	51	Father	Anorexia nervosa	17	female
E 11	Woman	57	Mother	Anorexia nervosa	18	female
E 12	Man	52	Father	Bulimia nervosa	17	female

E: interview.

**Table 2:** Semi-structured interview script.

Research Area	Interview Questions
Diagnostic feeling	How did you feel when you were informed of the diagnosis? How did you realize that your family member had an ED? Do you think you would have approached the problem differently today?
Family relations	4. What was your family relationship like before the diagnosis? 5. Do you consider that this family relationship has changed since the time of diagnosis? If so, how has it changed? 6. Have you noticed any impact on the coexistence within the family nucleus since the diagnosis was made? 7. What challenges or difficulties have you faced as a family since the diagnosis and how have you dealt with these changes together?
Assistance	8. Do you think that the resources/assistance provided by the socio-health services have been sufficient for the patient and the family? 9. How do you think support for these patients and their families could be improved?
Treatment	10. Do you consider the involvement of the family in the treatment of a patient with EDs to be important? 11. Did you feel involved in the treatment your family member received? 12. Have you been actively involved in treatment? If so, what has your experience been like in that process?

**Table 3:** Rigor criteria.

Criteria	Techniques and Procedures Used
Credibility	-Researcher triangulation: Each interview was analyzed by five researchers (E.G.C.-B., C.G.-M., A.T.-R., E.M.-M., and P.D.-H.) and two researchers with clinical and research experience in mental health (P.D.-H. and C.G.-N.). Team meetings were held to compare analyses and identify categories and themes with the rest of the team; -The analysis was carried out by five researchers and an external auditor (E.V.-V.); -Triangulation of data collection methods: Semi-structured interviews were conducted, and field notes were taken by the researchers; -Participant validation (member checking): Participants were offered the opportunity to review the audio recordings to confirm their experiences. No additional comments were made by any of the participants.
Transferability	-Detailed description of the study conducted: The characteristics of the researchers, participants, settings, sampling strategies, and data collection and analysis procedures were described in detail.
Reliability/Trustworthiness	-External researcher audit: An external researcher (E.V-V) assessed the research protocol, focusing on the methods applied and the study design.
Confirmability	-Researcher triangulation, member checking, and data collection triangulation were performed.

**Table 4:** Themes and categories.

	Themes (T)	Categories
**T1**	Diagnosis	Triggers Personality traits Behavioral changes Impact
**T2**	Family	Family relationship Marriage Change of role Role of the primary caregiver Involvement
**T3**	Resources	Scarce public resources Private costs Multidisciplinary team Nursing care
**T4**	Treatment	Approaching the situation Seeking assistance and coping Needs for improvement

## Data Availability

The data are available from the first author upon request.

## Supplementary Materials

The following supporting information can be downloaded at: https://www.mdpi.com/article/10.3390/nursrep14040302/s1. The supporting information can be reviewed in the COREQ Checklist document.

## Public Involvement Statement

There was no public involvement in any aspect of this research.

## Guidelines and Standards Statement

This manuscript was written following the COREQ guideline for qualitative research.
